# Prevention of Osteoporosis in the Ovariectomized Rat by Oral Administration of a Nutraceutical Combination That Stimulates Nitric Oxide Production

**DOI:** 10.1155/2019/1592328

**Published:** 2019-06-02

**Authors:** Rebecca A. Rajfer, Manuel Flores, Andrea Abraham, Eduardo Garcia, Natalhy Hinojosa, Mina Desai, Jorge N. Artaza, Monica G. Ferrini

**Affiliations:** ^1^Department of Orthopedic Surgery, Loma Linda University, Loma Linda, CA, USA; ^2^Department of Health and Life Sciences, Charles R. Drew University of Medicine and Science, Los Angeles, CA, USA; ^3^Perinatal Research Laboratory, Department of Obstetrics and Gynecology, Los Angeles Biomedical Research Institute at Harbor-UCLA Medical Center, Torrance, CA, USA; ^4^Department of Medicine, David Geffen School of Medicine at UCLA, Los Angeles, USA

## Abstract

Osteoporosis represents an imbalance between bone formation and bone resorption. As a result of low estrogen levels, it is markedly prevalent during menopause, thus making such patients susceptible to fractures. Both bone formation and resorption are modulated by nitric oxide (NO). Currently, there are no risk-free pharmaceutical prevention therapies for osteoporosis. COMB-4, a nutraceutical combination of Paullinia cupana, Muira puama, ginger, and L-citrulline, known to activate the NO-cGMP pathway, was reported to accelerate fracture healing in the rat. To determine whether COMB-4 could be effective in preventing menopausal osteoporosis, it was compared to estradiol (E2) in an ovariectomized (OVX) rat osteoporosis model. Nine-month-old female Sprague Dawley rats were divided into SHAM, OVX, OVX+E2, and OVX+COMB-4. After 100 days of treatment, bone mineral density (BMD) and bone mineral content (BMC) were measured by DXA scan. TRAP staining was performed in the femur and lumbar vertebrae. TRACP 5b and osteocalcin levels were assayed in the serum. MC3T3-E1 cells were differentiated into osteoblasts and treated with COMB-4 for one week in order to evaluate calcium deposition by Alizarin staining, cGMP production by ELISA, and upregulation of the nitric oxide synthase (NOS) enzymes by RT-PCR. OVX resulted in a decrease in BMD, BMC, and serum osteocalcin and an increase in serum TRACP 5b. Except for an increase in BMC with COMB-4, both E2 and COMB-4 reverted all bone and serum markers, as well as the number of osteoclasts in the vertebrae, to SHAM levels. Incubation of MC3T3-E1 cells with COMB-4 demonstrated an increase in the three NOS isoforms, cGMP, and calcium deposition. COMB-4 increased BMD in OVX rats by inhibiting bone resorption and increasing calcium deposition presumably via activation of the NO-cGMP pathway. It remains to be determined whether COMB-4 could be a potential nutraceutical therapy for the prevention of premenopausal bone loss.

## 1. Introduction

Osteoporosis, defined as a reduction in bone mass and a disruption of bone architecture, results in a decrease in bone integrity and an increase in fractures. Greater than two million osteoporotic fractures occur annually in the US [1]. One in every two women and one in every four men over age 50 will have an osteoporosis related fracture in their lifetime [2]. The major sites for such fractures are the vertebrae, hip, and wrist [3]. For those with hip fractures, there is an overall mortality of up to 33% [4–8], many are unable to walk independently at one year [9], more than half require assistance with daily living [9, 10], nearly 20% will require care in a long-term facility [4], and up to 42% fracture again within five years [11]. As such, the cost for treating patients with osteoporotic fractures represents a major burden to the entire health care system [2, 12].

Osteoporosis begins in the latter part of the third decade of life [13]. The integrity of the bone is maintained by a remodeling process in which the old bone is removed by osteoclasts and the new bone is formed by osteoblasts. Bone mass decreases when bone loss is greater than bone formation [14]. Therefore, in order to prevent osteoporotic fractures, prevention of bone resorption is paramount. Although age-related bone loss is multifactorial in both men and women, it is estrogen deficiency that appears to have a dominant role in both sexes [15–17]. Physiologically, the estrogen deficiency leads to an increase in osteoclast recruitment and a decrease in its apoptosis. However, the use of exogenous estrogen replacement has demonstrated deleterious side effects [18] and thus hormone replacement therapy for the prevention of osteoporosis is not universally recommended.

Nitric oxide (NO) is a signaling molecule that has been shown to be involved in bone function [19] and the therapeutic effects of estrogen on bone appear to be mediated by NO [20]. Its production by the inducible form of the nitric oxide synthase enzyme (iNOS) has been shown to be inhibitory to the osteoclast [19], while upregulation of NO either via its direct administration or via the administration of PDE-5 inhibitors appears to enhance fracture healing and bone regeneration [21, 22].

It was recently reported that an oral product consisting of the mixture of three natural products (Paullinia cupana, Muira puama, and ginger) together with the amino acid L-citrulline was capable of stimulating the production of iNOS, NO, and cGMP by the corporal smooth muscle cells of the rat [23]. It was then demonstrated in the same animal model that this nutraceutical, when given orally, was capable of stimulating iNOS production in bone, resulting in an enhancement of fracture healing greater than that observed with a PDE5 inhibitor [24].

Since it was assumed that the enhanced fracture healing observed with this oral product was most likely due to its effect on the osteoblast/osteoclast relationship, this prompted us to evaluate the potential efficacy of this natural combination product in the prevention of osteoporosis.

## 2. Materials and Methods

### 2.1. Experimental Animals

Thirty-two female Sprague Dawley 9-month-old retired breeder rats (B.W: 250±30g) from Envigo Laboratories (Livermore, CA) were used in the study. This protocol was approved by the Institutional Animal Care and Use Committee (IACUC) at Charles R. Drew University of Medicine and Science.

#### 2.1.1. Ovariectomy and SHAM Operations

All animals were treated the night before surgery with ibuprofen liquid gel capsules (0.4 mg/ ml) dissolved in drinking water. The animals underwent either ovariectomy (OVX; n = 24) or SHAM operation (SHAM; n = 8). Bilateral ovariectomy was performed as previously described [25] but under general isoflurane anesthesia (4-5% induction; 1.5-2% maintenance). For SHAM rats, the ovaries were exposed, examined, and then replaced into the abdominal cavity. In order to avoid pain, all animals were treated with subcutaneous administration of buprenorphine (0.05mg/kg B.W) for preoperative pain and every 8 to 12 hours for two days to prevent postoperative discomfort. Animals were housed in pairs. Additionally, all animals were assessed at least once daily to ensure that the incision site was free from infection and inflammation.

#### 2.1.2. Experimental Design

The day after surgery, animals were divided into four groups (n=8 per group), which consisted of a non-ovariectomized group (SHAM), a bilateral ovariectomized group (OVX), an OVX plus estradiol (E2) treated group (OVX+E2), and an OVX plus COMB-4 treated group (OVX+COMB-4). The SHAM and OVX groups were treated with the vehicle consisting of only peanut butter, DMSO, and water in a proportion of 1:0.1:10 ml, respectively. The OVX+E2 group was treated with vehicle containing estradiol valerate (0.8 mg/Kg. B.W.), dissolved in 0.1% dimethyl sulfoxide (DMSO). The OVX+COMB-4 group was treated with COMB-4, which comprised Paullinia cupana (45 mg/Kg B.W.), Muira puama (45 mg/Kg B.W.), ginger (45 mg/Kg B.W.), and L-citrulline (133 mg/kg B.W.) as previously described [24]. All animals received normal chow and water ad libitum. Vehicle and treatments, which were given orally by retrolingual administration [23], began the day after the surgery and the animals were treated for 100 days. The E2, L-citrulline, and DMSO were obtained from Sigma-Aldrich (St. Louis, MO). Paullinia cupana, Muira puama, and ginger were all obtained from Naturex (Hackensack, NJ).

### 2.2. DXA Scan Measurement

One day after the last treatment, all animals (n=32) underwent a noninvasive dual energy X-ray absorptiometry scan (DXA) using a software program for small animals (QDR 4500A; Hologic, Bedford, MA). Before scanning, the animals were anesthetized with ketamine/xylazine (45/6 mg/Kg. B.W.). The measurements with repositioning of the body were repeated three times to calculate the mean of bone mineral density (BMD), bone mineral content (BMC), fat mass, lean mass, lean mass+BMC, and total mass. The total mass was defined as the sum of the BMC, lean mass, and fat mass, and this was corroborated by the total body weight of the animal at the time of euthanasia.

After regaining consciousness and voluntary movement from anesthesia following the DXA scan, the animals were housed for three days and then euthanized by CO2 inhalation. Blood was then drawn by right cardiac atrial puncture immediately after death but prior to tissue collection.

### 2.3. Determination of TRAP Expression in Femur and Lumbar Vertebrae

Lumbar vertebrae (L4-5) and femora were dissected out by removing the fascia and muscle tissue. Bone specimens were fixed in 4% p-formaldehyde and decalcified in 10% formic acid for 7 days as previously described [24]. Specimens were then rinsed in phosphate buffer saline (PBS) and processed for paraffin embedding sections.

For the measurement of TRAP staining, cross sections of the body of the lumbar vertebra using the spinous process as landmark for selecting the anatomical matched sections were used. The femora were cut longitudinally, and the proximal epiphysis at the level of trochanteric line was used as a landmark to ensure that all sections were anatomically matched. Anatomically matched sections of the lumbar vertebra and femur were deparaffinized with CitriSolv (Fisher Sci, Hampton, NH) and rehydrated with decreasing concentrations of ethanol to distilled water. Sections were incubated with 0.2M Tris buffer (pH: 9) at 37°C for two hours. The sections were incubated in a prewarmed TRAP staining solution containing TRAP Basic Incubation Medium (Sodium Acetate Anhydrous L-Tartaric Acid), Fast Red Violet LB Salt, and Naphthol AS-MX Phosphate Substrate mix at 37°C for two hours. Sections were counterstained with hematoxylin for 15 seconds and rinsed quickly in distilled water. After checking for staining, the sections were dehydrated through graded alcohol and cleared with CitriSolv. Stained sections were mounted with Permount (Fischer Scientific, Hampton, NH). Pictures were taken with a Leica microscope (Leica Microsystems, Wetzlar, Germany) at 100x and 400x magnification. The number of TRAP-positive cells per field in the femur and lumbar vertebrae was quantified using ImagePro 7.1 software (Media Cybernetics, Silver Spring, MD).

### 2.4. Measurement of Serum Osteocalcin

Serum osteocalcin of the SHAM and treated animals was measured by ELISA using a Rat-MID Osteocalcin EIA assay kit (Immunodiagnostic Systems, IDS, UK) following the manufacturer's instructions.

### 2.5. Measurement of Serum Tartrate-Resistant Acid Phosphatase 5b by ELISA

The levels of rat tartrate-resistant acid phosphatase 5b (TRACP 5b) in serum of control and treated animals were measured by ELISA using a TRACP 5b assay kit (Immunodiagnostic Systems, IDS, UK) by incubating a specific monoclonal anti-rat TRACP 5b antibody monoclonal in an anti-mouse IgG-coated microtiter wells for 60 minutes at 20-24°C. After washing, standard, control, and serum samples were incubated in the wells by duplicates, and bound TRACP 5b activity was determined by adding a releasing reagent and a chromogenic substrate to develop color. The reaction was terminated and the absorbance of the reaction mixture was read at 405 nm in a microtiter plate reader. Absorbance is directly proportional to the activity of TRACP 5b present in the sample.

### 2.6. MC3T3-E1 Cell Culture

The MC3T3-E1 subclone 4 cell line, which is a preosteoblast cell line from C57BL/6 mouse calvarias, was obtained from American Type Culture Collection (Manassas, VA, USA) and cultured at 37°C in 5% CO_2_ atmosphere in Dulbecco's *α*-modified minimal essential medium (*α*-MEM); Gibco; Thermo Fisher Scientific, Inc., Waltham, MA, USA, supplemented with 10% heat inactivated fetal bovine serum (Sigma-Aldrich, Merck KGaA; Darmstadt, Germany) 100 U/ml penicillin and 100 *μ*g/ml streptomycin.

MC3T3-E1 cells were seeded at approximately 0.5–1.0×10^6^ cells per flask and were differentiated into osteoblasts using *α*-MEM supplemented with 50 *μ*g/mL L-ascorbic acid (Sigma-Aldrich,) and 10 mM *β*-glycerophosphate (Sigma-Aldrich,), which was replaced daily. Cells were differentiated for 10 days and were checked every day for contamination and proper differentiation.

#### 2.6.1. Mineralization Assay (Alizarin Red S Staining)

After differentiation, cells were treated with COMB-4 in ethanol (0.685mg/mL) on a daily basis for one week. To measure the calcium deposits in mineralized cells after the treatment, cells were washed twice with PBS, fixed in 4% paraformaldehyde for 30 minutes at room temperature, and washed again with distilled water. The cells were then stained for 30 minutes at room temperature using an Alizarin Red S solution pH 4.1-4.3 (Sigma-Aldrich) and washed three times with distilled water. Pictures were taken with a Leica microscope at low magnification (5X) to cover the entire well.

Mineralization was determined by measuring the red-stained area versus the nonstained area using ImagePro 7.1 software (Media Cybernetics, Silver Spring, MD). The results were expressed as the percentage of positive staining/ the total area of the well.

#### 2.6.2. Determination of cGMP

Differentiated MC3TC cells were incubated in a 6-well plate with vehicle and COMB-4 (0.685 mg/ml) for 24 hours. Incubation was stopped by aspirating the media and by adding 400 *μ*l HCL 0.1 M for 20 minutes, as previously described [26]. Cells were then scraped, homogenized by pipetting, and centrifuged at 1,000 g for 10 minutes. The supernatants were used for the determination of cGMP concentration by a colorimetric ELISA (Cayman Chemical Company, Ann Arbor, MI), following the manufacturer's instructions [26]. Fifty microliters of the standard dilutions and samples without acetylation were applied to a plate containing a rabbit antibody specific for cGMP that bounds to the wells coated with mouse anti-rabbit IgG. Binding was determined by competition with a cGMP tracer. After overnight incubation, the wells were washed, followed by incubation with Ellman's reagent. The enzymatic reaction product was determined by spectrophotometry at 405-nm absorbance and expressed as pmol/mg protein [26].

#### 2.6.3. Real-Time Quantitative PCR (qPCR)

Total RNA was extracted using Trizol Reagent (Invitrogen, Carlsbad, CA) and equal amounts (1 *μ*g) of RNA were reverse transcribed using High Capacity RNA-to-cDNA PCR kit (Applied Biosystems, Foster City, CA). Mouse gene PCR primer sets for nNOS, iNOS, and eNOS were obtained from SA Biosciences (Germantown, MD). The Power SYBR green PCR Master Mix (Applied Biosystems) was used with Step-One-Plus real-time PCR System (Applied Biosystems). The protocol included melting for 15 min at 95°C, 40 cycles of three-step PCR including melting for 15 s at 95°C, annealing for 30 s at 58°C, elongation for 30 s at 72°C with an additional detection step of 15 s at 81°C, followed by a melting curve from 55 to 95°C at the rate of 0.5°C per 10 s. The samples of 25 ng cDNA were analyzed in quadruplicate in parallel with RPLP1/3 controls; standard curves (threshold cycle vs. log pg cDNA) were generated by log dilutions of standard cDNA (reverse transcribed mRNA from differentiated osteoblast cells in growth media) from 0.1 pg to 100 ng. Experimental mRNA starting quantities were then calculated from the standard curves and averaged using SA Bioscience software. The ratios of marker experimental gene of the three NOS isoforms to that of RPLP1/3 mRNA were computed and normalized with control (untreated) samples as a ratio of 1.

### 2.7. Statistical Analysis

Values were expressed as mean ± SEM. The normal distribution of the data was established using the Wilk-Shapiro test. Multiple comparisons of the four groups were analyzed by a single factor analysis of variance (ANOVA), followed by post hoc comparisons with the Tukey test, according to the GraphPad Prism V 5.1. Differences were considered significant at p < 0.05. The use of eight animals per group was selected to obtain an 80% power at alpha 0.05. In the* in vitro* studies, experiments were repeated at least twice, and data from representative experiments are shown.

## 3. Results

### 3.1. Body Weight and Fat Mass following Treatment with Either E2 or COMB-4 for 100 Days following Ovariectomy

**Figure 1:**
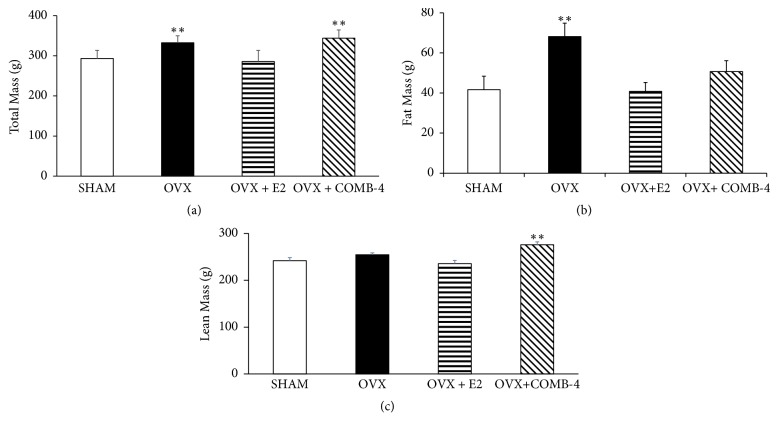
*Effect of 100 days treatment with E2 or COMB-4 on Total Body Mass, Fat Mass, and Lean Mass following OVX.* Whole body dual energy X-ray absorptiometry (DXA) scan performed 100 days following surgery and subsequent treatment with either vehicle (SHAM and OVX), E2 (OVX + E2) or COMB 4 (OVX + COMB-4).* Panel (a):* total body mass (g ±SEM); *∗∗* p < 0.01 with respect to SHAM and E2.* Panel (b):* total fat mass (g ± SEM); *∗∗* p < 0.01; with respect to SHAM, E2, and COMB-4.* Panel (c):* total lean mass (g± SEM) *∗∗*p<0.01 with respect to SHAM, OVX, and E2. n = 8 per group.

In order to determine whether treatment with either E2 or COMB-4 can influence body weight in OVX rats, total mass (Figure 1(a)), fat mass (Figure 1(b)), and lean mass (Figure 1(c)) were determined following 100 days of treatment after bilateral ovariectomy. The total mass of the OVX-only animals was significantly increased compared to the SHAM group (p<0.01), and this was restored to SHAM levels by E2 (E2 + OVX) (Figure 1(a)), while treatment with COMB-4 (OVX + COMB-4) did not restore total mass to the SHAM level as seen in Figure 1(a). Figures 1(b) and 1(c) show that COMB-4 treatment for 100 days in OVX rats did not increase fat mass but increased the lean mass.

### 3.2. Treatment with E2 or COMB-4 for 100 Days following Ovariectomy Prevents Reduction in Bone Mineral Density and Bone Mineral Content

**Figure 2:**
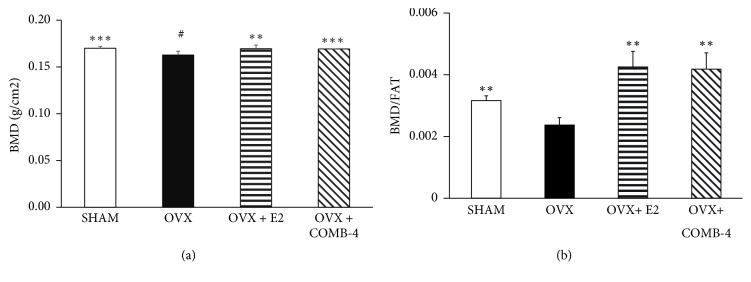
*Effect of 100 days treatment with E2 or COMB-4 on Bone Mineral Density following OVX. Panel (a):* bone mineral density (BMD; g/cm2 + SEM) determined by DXA scan in SHAM, OVX, E2(OVX+E2), and COMB-4 (OVX+COMB-4). *∗∗∗* p < 0.001; *∗∗* p < 0.01 with respect to OVX; # p < 0.05 respect to SHAM.* Panel (b):* BMD normalized by fat content in SHAM. OVX, E2 (OVX+E2), and COMB-4 (OVX+COMB-4). *∗∗* p < 0.01 with respect to OVX. n = 8 per group.

Whole body bone mineral density (BMD) was determined in all four groups by DXA scan 24 h following 100 days of treatment after bilateral ovariectomy (Figure 2(a)). The OVX group, which only received vehicle for 100 days, showed a significantly lower BMD with respect to the SHAM group (p < 0.05) while treatment with either E2 or COMB-4 prevented the loss of BMD as seen in the OVX-only animals (p < 0.01 and p < 0.001, respectively). When BMD was normalized to the fat mass due to interference of fat in the BMD measurements [27, 28], a more pronounced increase in BMD was observed in the COMB-4 and E2 treated groups, in contrast with the decrease in BMD observed in the OVX group (Figure 2(b)).

**Figure 3:**
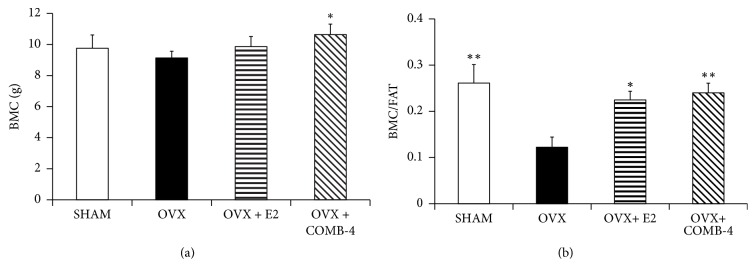
*Effect of 100 days treatment with E2 or COMB-4 on Bone Mineral Content following OVX. Panel (a):* bone mineral content (BMC; g + SEM) determined by DXA scan in SHAM, OVX, E2 (OVX+E2), and COMB-4 (OVX+COMB-4). *∗∗* p < 0.01 respect to OVX; # p < 0.05 respect to SHAM.* Panel (b):* BMD normalized by fat content in SHAM. OVX, E2 (OVX+E2), and COMB-4 (OVX+COMB-4). *∗* p < 0.05; *∗∗*p < 0.01 with respect to OVX. n = 8 per group.

The bone mineral content (BMC) of the animals was also analyzed by DXA scan.  Figure 3(a) shows that treatment with COMB-4 for 100 days following ovariectomy resulted in a significant increase in BMC when compared to the OVX-only group, thus suggesting that the increase in total body weight observed in the COMB-4-treated group (Figure 1(a)) was due to an increase in BMC and/or lean mass (Figure 1(c)).  Figure 3(b) shows the results of normalizing the BMC to fat mass as was done for the BMD. The OVX group showed the lowest BMC/fat ratio. However, treatment with either E2 or COMB-4 restored this ratio to that seen in the SHAM group.

### 3.3. Effect of E2 or COMB-4 on Serum Biochemical Markers after Ovariectomy

**Figure 4:**
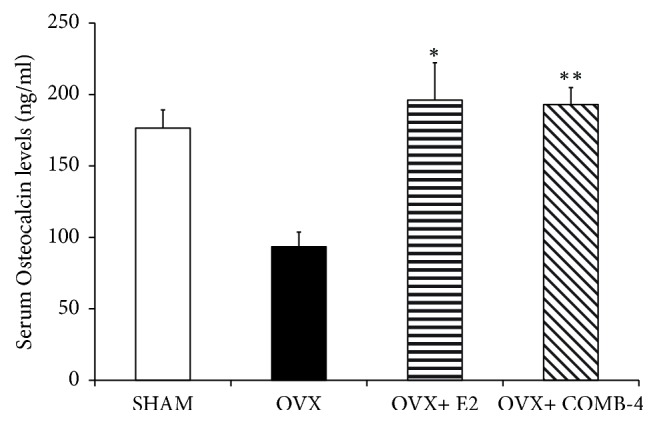
*Level of Serum Osteocalcin after 100 days treatment with E2 or COMB-4 following OVX.* Osteocalcin (ng/ml + SEM) in serum was determined by ELISA in SHAM, OVX, OVX+E2, and OVX+ COMB-4 groups. *∗*p < 0.05; *∗∗*p < 0.01 with respect to OVX. n = 8 per group.

Osteocalcin, a marker of osteoblast activity, was measured in the serum after 100 days of treatment. A significant (p < 0.05) reduction in osteocalcin levels was observed in the OVX animals in comparison to the SHAM group (Figure 4). Treatment with either E2 or COMB-4 prevented the marked reduction in osteocalcin levels seen in the OVX animals (p < 0.05).

**Figure 5:**
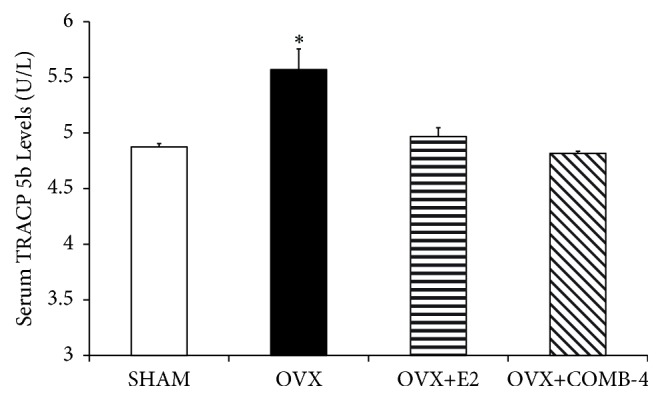
*Level of serum tartrate-resistant acid phosphatase (TRACP 5b) after 100 days treatment with E2 or COMB-4 following OVX.* Serum TRACP 5b (U/L + SEM) levels were determined by ELISA. *∗*p < 0.05 with respect to SHAM, OVX+E2, OVX+COMB-4. n = 8 per group.

The levels of serum tartrate-resistant acid phosphatase (TRACP 5b), a marker of osteoclast activity, were also measured at 100 days in the four groups (Figure 5). OVX significantly increased TRACP 5b levels by 20% (p < 0.05) with respect to that of the SHAM group, while treatment with either E2 or COMB-4 following OVX was not significantly different from those of the SHAM group (p = 0.5337 and p=0.9931, respectively).

### 3.4. Expression of TRAP in the Lumbar Vertebrae and Femur after Treatment with E2 or COMB-4 to OVX Rats

**Figure 6:**
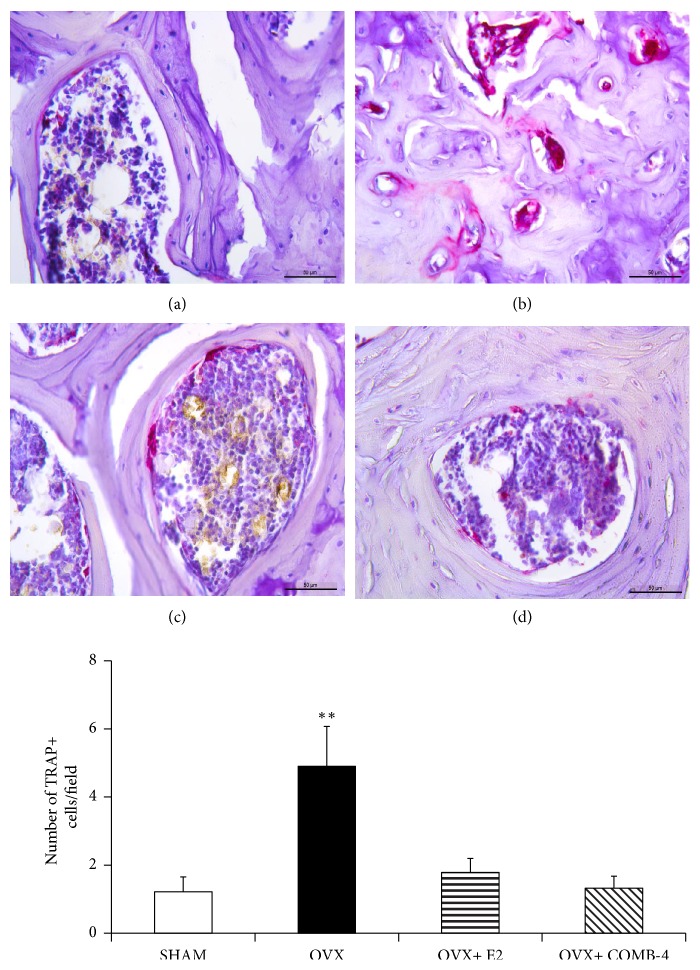
*Tartrate acid phosphatase (TRAP) staining in lumbar vertebrae.* Five *μ*m paraffin embedded sections of L4-5 vertebrae were stained with prewarmed TRAP staining solution, containing Fast Red Violet LB Salt and Naphthol AS‐MX Phosphate Substrate.* Top Panel:* representative images of positive trap cells in (a): SHAM, (b): OVX, (c): OVX+E2, and (d): OVX+COMB-4 after 100 days treatment. TRAP+ cells are stained in pink/violet. Sections were counterstained with hematoxylin to observe the cell nuclei. Magnification 400X; Bar: 50 um.* Bottom Panel:* number of TRAP-positive staining cells per field. *∗∗*p < 0.05 respect to SHAM, OVX+E2, OVX+COMB-4. n = 8 per group.

Expression of TRAP as a marker of osteoclast activity in paraffin embedded sections was performed to quantify the number of TRAP-positive cells in lumbar vertebrae and femur.  Figure 6 top panel shows representative photographs in the lumbar vertebra for (a) SHAM; (b) OVX; (c) OVX+E2; and (d) OVX+COMB-4. An increase in the expression of TRAP-positive cells was observed in the OVX group (Figure 6(b)).  Figure 6-bottom panel shows the results of the quantification of the number of positive cells per field in the spongy bone of the lumbar vertebra. A fivefold increase in the number of TRAP-positive cells was observed in the OVX group when compared to the SHAM group (p<0.05). Treatment with E2 or COMB-4 to OVX rats prevents any increase in the number of these TRAP-positive cells as seen in the OVX group.

**Figure 7:**
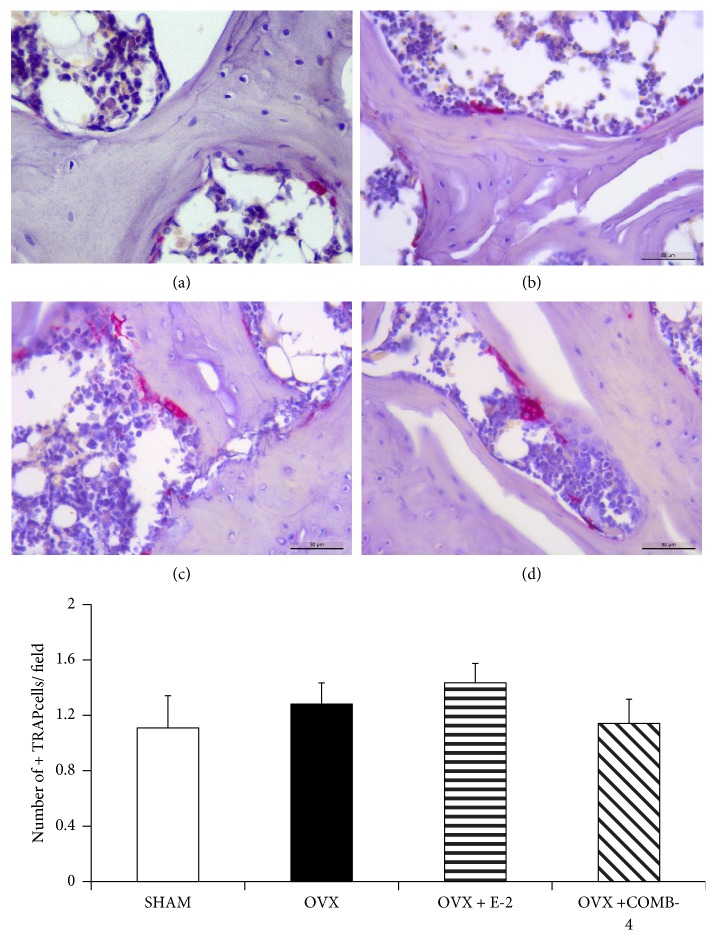
*Tartrate acid phosphatase (TRAP) staining in femur.* Paraffin embedded sections of proximal epiphysis of the femora were stained with prewarmed TRAP staining solution, as in Figure 6.* Top Panel:* representative images of positive trap cells in (a): SHAM, (b): OVX, (c): OVX+E2, and (d): OVX+COMB-4 after 100 days of treatment. TRAP+ cells are stained in pink/violet. Sections were counterstained with hematoxylin to observe the cell nuclei. Magnification 400X; Bar: 50 um.* Bottom Panel:* number of TRAP-positive staining cells per field. No significant difference was observed among the groups. n = 8 per group.

Figure 7 top panel shows representative slides of the TRAP staining in the proximal femur epiphyses at the level of the trochanter of SHAM (Figure 7(a)); OVX, (Figure 7(b)); OVX+E2, (Figure 7(c)); and OVX+COMB-4, (Figure 7(d)). Unlike the lumbar vertebra, the expression of TRAP in the proximal epiphysis of the femur did not show a significant difference among the groups (Figure 7-bottom panel).

### 3.5. Effect of COMB-4 on Calcium Deposition in an Osteoblast Cell Line

**Figure 8:**
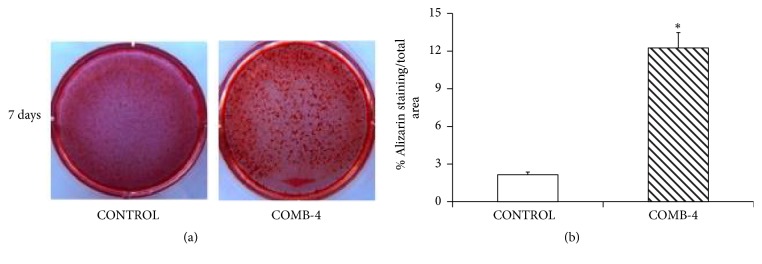
*Effect of the incubation of COMB-4 on calcium deposition in differentiated osteoblastic MC3T3-E1 cell line. Panel (a):* representative images of MC3T3-E1 cell line differentiated for ten days into osteoblasts and incubated with or without COMB-4 for one week. Calcium deposition after COMB-4 incubation done by Alizarin red S staining.* Panel (b):* quantitative measurements of Alizarin red S staining of the differentiated MC3T3-E1. Results are expressed as percent of positive Alizarin red area/total area and represent the mean ± SEM of three experiments. *∗*p < 0.05 respect to the control group.

In order to determine whether the effect observed with COMB-4 in preserving BMD in OVX rats was due to a stimulation of calcium deposition, an experiment utilizing a preosteoblastic cell line, MC3T3-E1, was designed. Cells were differentiated into osteoblasts with ascorbic acid and *β*-glycerophosphate and then incubated with or without COMB-4 for 1 week.  Figure 8(a) shows representative wells of calcium deposition measured by alizarin red staining in control and COMB-4.  Figure 8(b) shows that after 1-week incubation with COMB-4, a sixfold increase of the positive Alizarin staining area with respect to the control group was observed.

### 3.6. COMB-4 Upregulates NO-cGMP Pathway in Differentiated MC3T3-E1 Cells

In order to determine whether COMB-4 acts similar to E2 in activating the NO-cGMP pathway, the expression of cGMP was determined in the differentiated MC3T3-E1 cell line.

**Figure 9:**
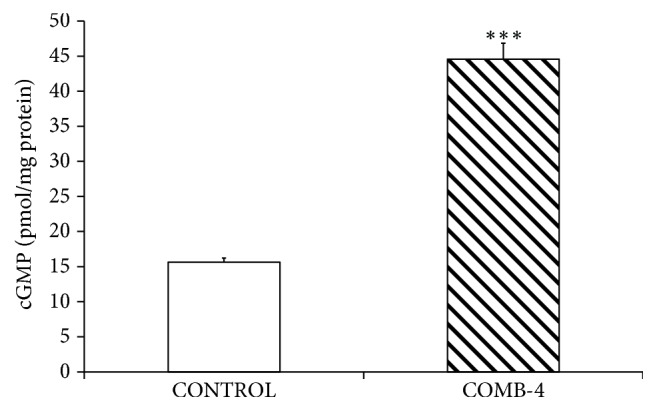
*Effect of the incubation of COMB-4 on cGMP expression in differentiated MC3T3-E1 cell line.* After MC3T3-E1 cells were differentiated with ascorbic acid and *β*-glycerophosphate for seven days, osteoblast cells were incubated for 24 hours with or without COMB-4. cGMP expression was determined by ELISA. Results are expressed as pmol/mg of protein and represent the mean ± SEM of four experiments done in duplicate. *∗∗∗*p < 0.01 with respect to control.

**Figure 10:**
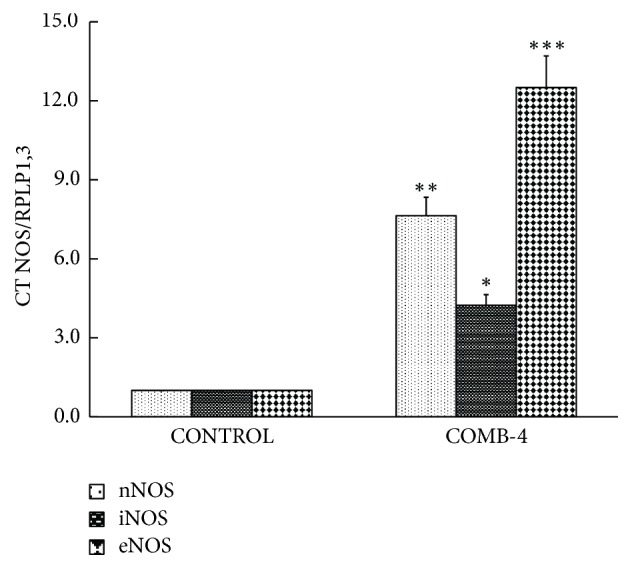
*Effect of the incubation of COMB-4 on nNOS, iNOS, and eNOS mRNA expression in differentiated MC3T3-E1 cell line.* Differentiated MC3T3-E1 cells were incubated with or without COMB-4 for 48 hours. mRNA expression for nNOS, eNOS, and iNOS was determined by qPCR. Results were expressed as x-fold increase with respect to the control of three experiments done in triplicate. *∗*p<0.05; *∗∗*p<0.01; *∗∗∗*p<0.001 with respect to control.

Figure 9 shows that the incubation of differentiated cells with COMB-4 for 24 hours produced a threefold increase in the expression cGMP indicating activation of the NO-cGMP pathway by COMB-4 in this cell line. In order to determine whether any of the three NOS isoforms is upregulated within the osteoblast by COMB-4, the expression of these three individual isoforms was measured in the MC3T3-E1 cells. COMB-4 induced a 12-fold increase in the mRNA expression of eNOS, a 7-fold increase in nNOS, and a 4-fold increase in iNOS (Figure 10).

## 4. Discussion

This study in the adult ovariectomized and presumably estrogen deficient rat demonstrates that the daily treatment with the nutraceutical combination, COMB-4, replicates the same effects that exogenous estrogen has on this animal model. These include (1) an increase in BMD and BMC as measured by DXA scan, (2) an increase in serum osteocalcin, and (3) a decrease both in the serum 5b TRAPc as well as the number of TRAP-positive cells in the lumbar vertebrae. This decrease in 5b TRAP in both the serum and bone is known to occur with estrogen deficiency suggesting that besides stimulating bone formation, COMB-4 may also be capable of inhibiting osteoclastic activity. In addition, incubation of differentiated MC3TC cells with COMB-4 resulted in an increase in all three NOS isoforms, cGMP expression and calcium deposition as determined by Alizarin staining.

The high prevalence of osteoporosis in the postmenopausal period is presumably due to the detrimental effect that the associated decrease in estrogen production has on bone mass and strength [14]. It is accepted that estrogen's effect on bone is mediated via NO [20, 29]. In bone, NO is synthesized by the NOS enzymes located either within the osteocytes, osteoblasts, and/or osteoclasts [30–32]. The primary function of NO in any tissue is to stimulate the intracellularly located guanylyl cyclase enzyme to form the second messenger, cGMP, which then stimulates a series of chemical reactions within the cell to accomplish its inherent function. Stimulation of the NO-cGMP pathway will inhibit apoptotic activity within the osteoblasts/osteocytes while simultaneously stimulating apoptotic activity within the osteoclasts [19–21].

While it is known that estrogen replacement reverses those osteoporotic characteristics associated with estrogen deficiency, its use is associated with potentially severe side effects such as the development of breast cancer and thromboembolic episodes [18, 33, 34]. As such, their clinical usefulness in this setting has been severely limited. Selective estrogen receptor modulators (SERMs) have also been tried as a treatment for postmenopausal osteoporosis because they have a lower breast cancer risk than that seen with estrogen itself, but the SERMs still carry the risk of developing thromboembolic episodes [35, 36]. This has relegated the bisphosphonates for the most part as the primary current treatment of postmenopausal osteoporosis but these too have a significant risk profile [36].

It therefore appears as if the solution to this dilemma of estrogen deficiency-induced osteoporosis is the use of a nonestrogenic product that will stimulate the NO-cGMP pathway within the bony architecture in order to counteract the loss of bone mass and bone strength. In keeping with that concept, various nonestrogenic products that either contain NO or release NO directly into the circulation have been tried to arrest postmenopausal osteoporosis but the results have been unconvincing [37–39]. COMB-4 is a natural product that was recently developed as a way to increase the production of NO and cGMP within the vascular smooth muscle cells [26]. It was shown that this stimulation of the NO-cGMP pathway in these vascular smooth muscle cells was mediated via the upregulation of iNOS within the vascular smooth muscle cell itself [26]. When the compound was used clinically in men [40], its side effect profile was relegated only to a complaint of a ginger aftertaste. This was not surprising since the use of ginger, Paullinia cupana, and/or Muira puama by itself has been reported previously to have a safe profile [41, 42].

One limitation of this study is that hormone levels were not measured in these animals. However, it seems unlikely that COMB-4 would be capable of stimulating estrogen since the animals that received COMB-4 were ovariectomized and there have been no reports in the literature of any estrogenic effects seen with any of these four constituents of COMB-4 [41, 42]. Another limitation of this study is that we also did not test bone strength, but the BMD and BMC data in those animals treated with COMB-4 appear very similar to that of the SHAM group. Finally, we did not measure the receptor of activated nuclear factor kappa-B ligand (RANKL) and its antagonist, osteoprotegerin, two cytokines that are involved in osteoclastic function that may also be modulated by COMB-4 [43]. Further investigation will elucidate the role of COMB-4 in this pathway.

In summary, COMB-4 appears to be capable of reproducing the same antiosteoporotic effects of estrogen, but without its side effects. Based on our original* in vivo* study that demonstrated COMB-4 enhanced fracture healing, together with this current study showing a proosteoblastic and antiosteoclastic effect of COMB-4, further investigation of COMB-4 may demonstrate it is a safe and effective preventive treatment for bone loss in women with estrogen deficiency.

## Data Availability

The DXA scans, histochemistry, bone serum markers, RT- PCR, and cGMP data used to support the findings of this study are available from the corresponding author upon request. The corresponding author takes responsibility for the integrity and availability of the data analysis.
